# Supplementary material for: Lola regulates Drosophila adult midgut homeostasis via non-canonical hippo signaling
Source: eLife. 2020 Jan 14;9:e47542. doi: 10.7554/eLife.47542 (PMC7299341; doi:10.7554/eLife.47542)
Supplement: Transparent reporting form [file elife-47542-transrepform.docx]

***eLife’s* transparent reporting form**

We encourage authors to provide detailed information *within their submission* to facilitate the interpretation and replication of experiments. Authors can upload supporting documentation to indicate the use of appropriate reporting guidelines for health-related research (see [EQUATOR Network](http://www.equator-network.org/%20)), life science research (see the [BioSharing Information Resource](https://biosharing.org/)), or the [ARRIVE guidelines](http://www.plosbiology.org/article/info:doi/10.1371/journal.pbio.1000412) for reporting work involving animal research. Where applicable, authors should refer to any relevant reporting standards documents in this form.

If you have any questions, please consult our Journal Policies and/or contact us: [editorial@elifesciences.org](mailto:editorial@elifesciences.org).

**Sample-size estimation**

- You should state whether an appropriate sample size was computed when the study was being designed
- You should state the statistical method of sample size computation and any required assumptions
- If no explicit power analysis was used, you should describe how you decided what sample (replicate) size (number) to use

Please outline where this information can be found within the submission (e.g., sections or figure legends), or explain why this information doesn’t apply to your submission:

No power analysis was performed in this study. Sample size (n) for each experiment is indicated in the figure legend.

For p-H3^+^ cell number quantitation (Figure 1E, Figure 1-figure supplement 1E, Figure 2C, Figure 2H, Figure 2-figure supplement 1C, Figure 2-figure supplement 1F, Figure 4D, Figure 5G, Figure 7A, Figure 7-figure supplement 1E, Figure 6-figure supplement 3E), at least 10 guts from indicated genotype were dissected and subjected to confocal analysis.

For Dl^+^ cell number per clone quantification (Figure 1I), at least 10 guts from indicated genotype were dissected and subjected to confocal analysis.

For clone size quantitation (Figure 1N, Figure 1-figure supplement 1O, Figure 7F, Figure 7-figure supplement 2D), all the clones in midguts from 10 guts of indicated genotype were calculated.

For adult wing size quantification (Figure 1-figure supplement 1J), at least 10 flies from indicated genotype were analysed.

For the quantification of indicated protein signal intensity (Figure 5F, Figure 6I), 50 cells from indicated view with GFP^+^ or GFP^-^ were quantified using the Leica AF Lite system.

**Replicates**

- You should report how often each experiment was performed
- You should include a definition of biological versus technical replication
- The data obtained should be provided and sufficient information should be provided to indicate the number of independent biological and/or technical replicates
- If you encountered any outliers, you should describe how these were handled
- Criteria for exclusion/inclusion of data should be clearly stated
- High-throughput sequence data should be uploaded before submission, with a private link for reviewers provided (these are available from both GEO and ArrayExpress)

Please outline where this information can be found within the submission (e.g., sections or figure legends), or explain why this information doesn’t apply to your submission:

Quantitative real-time PCR analysis and ChIP analysis were performed in biological triplicates. Each biological replicate contained three technical replicates. The technical replicates were averaged to obtain each biological replicate value. No outliers were excluded from the data.

Cell transfection, Co-IP and Western blot were performed in biological triplicates.

For all the fly experiments, three independent crosses were performed for each genotype as biological triplicates.

**Statistical reporting**

- Statistical analysis methods should be described and justified
- Raw data should be presented in figures whenever informative to do so (typically when N per group is less than 10)
- For each experiment, you should identify the statistical tests used, exact values of N, definitions of center, methods of multiple test correction, and dispersion and precision measures (e.g., mean, median, SD, SEM, confidence intervals; and, for the major substantive results, a measure of effect size (e.g., Pearson's r, Cohen's d)
- Report exact p-values wherever possible alongside the summary statistics and 95% confidence intervals. These should be reported for all key questions and not only when the p-value is less than 0.05.

Please outline where this information can be found within the submission (e.g., sections or figure legends), or explain why this information doesn’t apply to your submission:

Statistical analysis was performed using student’s T-test. Values are shown as mean ± SEM and p-values are indicated in the figure legends.

(For large datasets, or papers with a very large number of statistical tests, you may upload a single table file with tests, Ns, etc., with reference to sections in the manuscript.)

**Group allocation**

- Indicate how samples were allocated into experimental groups (in the case of clinical studies, please specify allocation to treatment method); if randomization was used, please also state if restricted randomization was applied
- Indicate if masking was used during group allocation, data collection and/or data analysis

Please outline where this information can be found within the submission (e.g., sections or figure legends), or explain why this information doesn’t apply to your submission:

No group allocation was used in this study.

**Additional data files (“source data”)**

- We encourage you to upload relevant additional data files, such as numerical data that are represented as a graph in a figure, or as a summary table
- Where provided, these should be in the most useful format, and they can be uploaded as “Source data” files linked to a main figure or table
- Include model definition files including the full list of parameters used
- Include code used for data analysis (e.g., R, MatLab)
- Avoid stating that data files are “available upon request”

Please indicate the figures or tables for which source data files have been provided:

Figure 1-source data 1

Source data for Figure 1E, Figure 1I, Figure 1N, Figure 1-supplement 1F, Figure 1-supplement 1K, and Figure 1-supplement 1P

Figure 2-source data 1

Source data for Figure 2C, Figure 2H, Figure 2-supplement 1D, and Figure 2-supplement 1G

Figure 4-source data 1

Source data for Figure 4D

Figure 5-source data 1

Source data for Figure 5F and Figure 5G

Figure 6-source data 1

Source data for Figure 6J

Figure 7-source data 1

Source data for Figure 7A, Figure 7F, Figure 7-supplement 1E, Figure 7-supplement 2D, and Figure 7-supplement 3E
